# Non-acid reflux and sleep apnea: the importance of drug induced sleep endoscopy

**DOI:** 10.1186/s40463-021-00526-w

**Published:** 2021-06-30

**Authors:** Carlos O’Connor-Reina, Jose Maria Ignacio Garcia, Peter Baptista, Maria Teresa Garcia-Iriarte, Carlos Casado Alba, Monica Perona, Paz Francisca Borrmann, Laura Rodriguez Alcala, Guillermo Plaza

**Affiliations:** 1 Otorhinolaryngology Department, Hospital Quiron Salud Marbella, Marbella, Spain; 2 Otorhinolaryngology Department, Hospital Quiron Salud Campo de Gibraltar, Cádiz, Spain; 3 Pulmonology Department, Hospital Quiron Salud Marbella, Marbella, Spain; 4 Pulmonology Department, Hospital Quiron Salud Campo de Gibraltar, Cádiz, Spain; 5 Otorhinolaryngology Department, Clinica Universitaria de Navarra, Pamplona, Spain; 6 Otorhinolaryngology Department, Hospital Universitario de Valme, Sevilla, Spain; 7 Clinica Universitaria de Navarra, School of Medicine, Pamplona, Spain; 8 Digestive Department, Hospital Quironsalud Marbella, Marbella, Spain; 9 Phonoaudiology Unit. Otorhinolaryngology Department Hospital Universitario Italiano Buenos Aires, Buenos Aires, Argentina; 10 Otorhinolaryngology Department, Hospital Universitario de Fuenlabrada & Hospital Sanitas la Zarzuela. Universidad Rey Juan Carlos, Madrid, Spain

**Keywords:** Obstructive sleep apnea, Nonacid reflux disease, Multichannel impedanciometry, Epiglottis, Continuous positive airway pressure, Drug-induced sleep endoscopy

## Abstract

**Background:**

We present the first case of a patient with obstructive sleep apnea syndrome (OSA), where drug induced sleep endoscopy was helpful to suspect a non-acid reflux disease and showed an improvement in a swollen epiglottis after treatment. Patient ameliorated significantly his disease only with medical therapy.

**Case presentation:**

A 54-year-old man without significant anatomical findings with obstructive sleep apnea syndrome and non-acid gastroesophageal reflux disease (GERD) disease whose Apnea- hypopnea index (AHI) was significantly reduced with the intake of 500 mg of sodium alginate twice a day for 6 months. Conventional digestive tests such as esophagoscopy and simple- and double-channel 24-h pH-metry suggested mild GERD. Conventional proton-pump inhibitor treatment with pantoprazole (40 mg daily) was started without any improvement in his sleep. Multichannel intraluminal 24-h impedanciometry indicated the presence of severe pathological GER of gaseous origin. The patient’s AHI decreased from 25.3 at baseline to 8 after treatment with sodium alginate. A drug-induced sleep endoscopy study showed the changes before and after this treatment and was helpful for the diagnosis.

**Conclusions:**

Thus, medical treatment can be a therapeutic option in some patients with OSA. Multichannel 24-h impedanciometry should be performed when nonacid GERD is suspected.

**Supplementary Information:**

The online version contains supplementary material available at 10.1186/s40463-021-00526-w.

## Background

Gastroesophageal reflux disease (GERD) can be categorized as acid and non-acid types. Non-acid reflux is defined as GERD episodes resulting in an esophageal pH drop to ~ 4.0. It is associated with refractory reflux symptoms in GERD with proton-pump inhibitor (PPI) failure and other extraesophageal symptoms, including coughing [1]. A laryngopharyngeal reflux (LPR) is defined as GERD reaching the laryngopharynx. It is supposed to cause inflammation in the airway and lead to respiratory symptoms. The exact role of reflux in the pathogenesis of obstructive sleep apnea syndrome (OSA) is still unclear, especially for non-acid reflux and LPR [2]. We present the first documented case of a patient with OSA where the epiglottis inflammatory changes after using gastric mucosal protector have been documented with a drug-induced sleep endoscopy (DISE) and correlated with a significant improvement in his apnea-hypopnea index (AHI).

## Case presentation

A 54-year-old man was referred from our Department of Pulmonology to our Department of Otorhinolaryngology complaining of continuous positive airway pressure (CPAP) device intolerance. He was not able to use it more than 3 h per night, despite changing masks and improving humidity. He only takes tadalafil 20 mg for erectile dysfunction and showed no other comorbidities. His wife did not complain about his snoring. His main concern was a sensation of suffocation during the night, as well as when lying on his back. His body mass index (BMI) was 23.1 m/kg^2^. Laboratory polysomnography (PSG) showed 299 snore events per hour, an AHI of 25.3, a saturated oxygen level (Sat O_2_) minimum of 74%, and an oxygen desaturation index (ODI) of 20.2. Awake rhinofibrolaryngoscopy did not show any anatomical findings that explained the CPAP intolerance showing an epiglottis with normal shape and appearance. In this case, our clinical protocol established the recommendation to perform a DISE. It was done following the European position paper protocol using the VOTE classification in the operating room, starting with the patient in supine position. For sedation, 2% propofol syringe infusion pump with target-controlled infusion (TCI) was used, with a target concentration of 2 ng/ml if progressive increases were requiered: 0.2–0.5 ng / ml. Sedation level was monitored using bispectral index (BIS) (BIS Quatro®. Covidien ILc. MA. USA). When the patient was asleep and actively snoring (BIS between 70 and 50), video-flexible endoscopy (TGH Endoscopia. MACHIDA ENT-30PIII. Spain) was used to assess the upper airway to visualize the site of collapse in real time and recording equipment. During DISE, the head was first turned to the right, then to the left and, finally, the mandibular advance maneuver was performed. The findings were observed for a minimum of two cycles in each segment and for each maneuver.

**Fig. 1:**
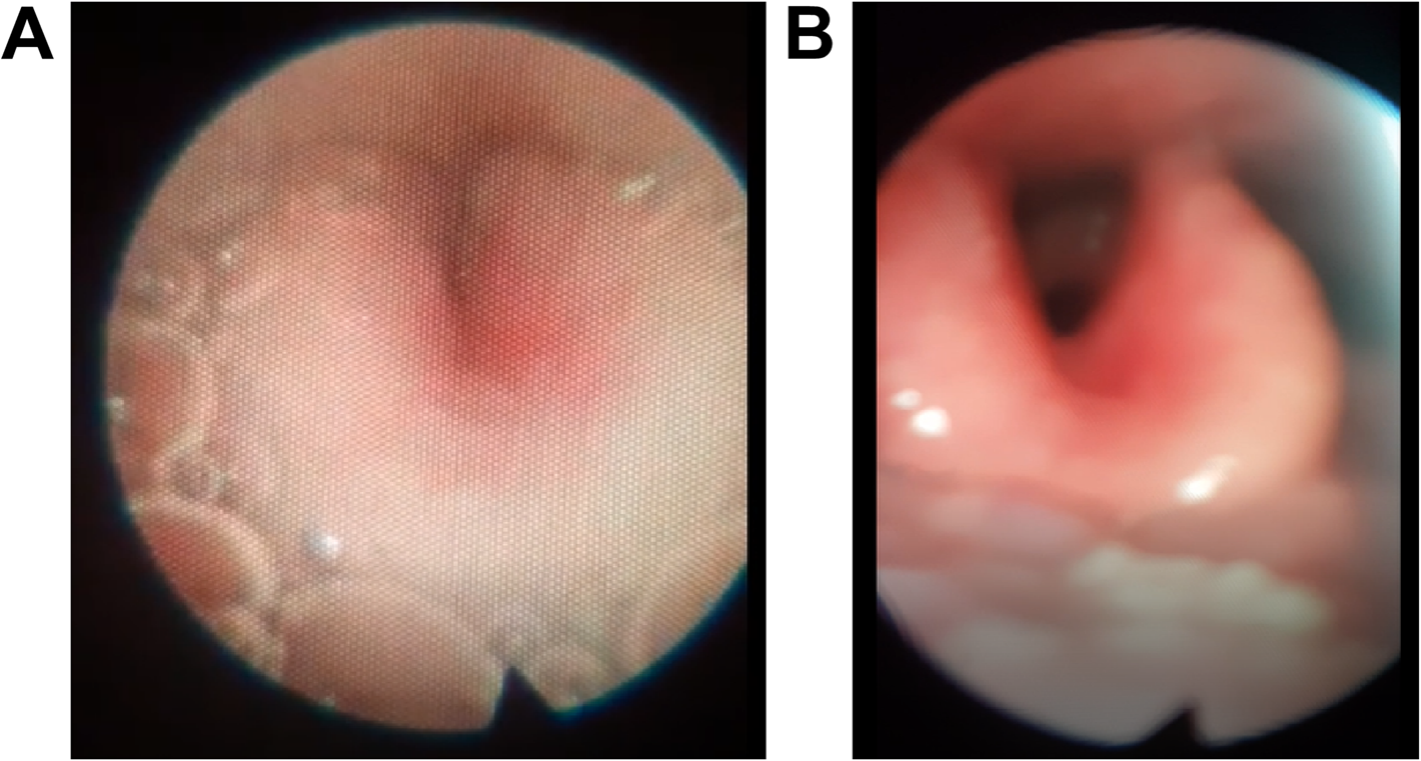
Epiglottis showing changes before (**A**) and after (**B**) 6 months of treatment with 500 mg sodium alginate daily


DISE was done without adverse incident and revealed significant swelling of the epiglottis (Supplementary Video 1). A severely obstructed airway surrounded by fluid compatible with LPR was found (Fig. 1A). The VOTE classification [3] was V1O1T1E2AP, which improved with the Esmarch maneuver.

**Additional file 1: Video S1.** Drug-induced sleep endoscopy showing active GER with a swelling epiglottis blocking the airway.

**Fig. 2:**
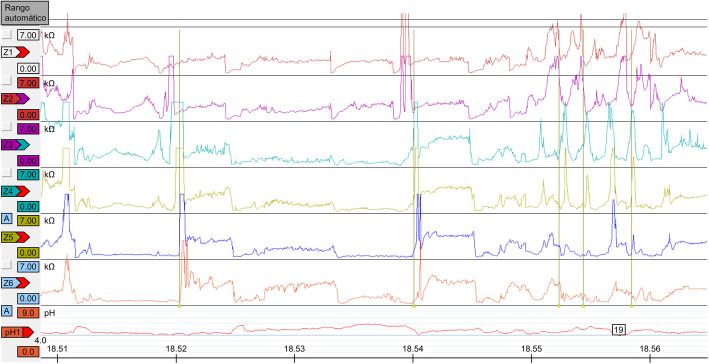
Multichannel impedanciometry (Z1-Z6) 24 h recording with multiple episodes of GER due to non acid reflux (gas). Ph1 showed peaks of non acid reflux (Ph > 4). Channels Z1 to Z6 showed the chapters where there is an increase of the impedance due to gas


With this diagnosis, the patient was offered a mandibular advancement device, but rejected it for economic reasons. He was referred to our Department of Gastroenterology. A PPI, pantoprazole (40 mg daily), was prescribed. Conventional esophagoscopy and single-channel pH-metry confirmed grade A esophagitis with mild GERD. Two-channel 24-h pH-metry indicated excellent suppression of gastric acid production with the PPI. Patient was recommended to avoid heavy meals for dinner and to raise the head of the bed for sleeping. The patient continued taking pantoprazole for 3 months but without improvement in his sleep symptoms, so he stopped the PPI treatment. During this time, he asked us to perform an epiglottectomy, but we rejected this option because non-acid reflux had not been discounted. Multichannel impedanciometry with 24-h pH-metry was performed and indicated severe alkaline GERD, mainly of gaseous origin (Fig. 2). Non-acid reflux therapy was started with sodium alginate (500 mg twice daily), and his sleep symptoms improved. After 6 months, a control PSG was performed. The AHI had decreased to 8, the Sat O_2_ min had improved to 87%, and the ODI had declined to 7.7 and snore events decline to 48 per hour. There was no change in his BMI. We performed a new DISE to evaluate the anatomical changes and this showed complete normality, with a VOTE classification of V1O1T1EO (Fig. 1B; Supplementary Video 2).

**Additional file 2: Video S2.** After 6 months of therapy with 500 mg sodium alginate daily. The epiglottis had recovered its normal shape.

Treatment with CPAP devices may aggravate airway obstruction in patients with a collapsed epiglottis [4]. Surgery in these cases could be an option [5], but it is always essential to evaluate the reason for collapse. In our case, the initial findings suggested a potential association with GERD, and DISE was mandatory for a complete evaluation [6]. A conventional ENT examination did not show any pathological findings of the epiglottis that were visualized during DISE.

## Discussion

There is great controversy on the possible link between GERD and OSA [7–9]. Some authors have considered that these diseases lack significant association, especially when non-acid GERD is diagnosed [2],. while other authors have shown such relation, especially after multichannel intraluminal impedance pH testing is done [10–12].

However there are few studies published about this matter, because the gold standard procedure to diagnose non-acid reflux, is the multichannel intraluminal impedance ph testing and this is expensive, invasive and not well tolerated in all the patients [13].

A recent metanalysis [14] has shown an overall incidence of 45,2% of LPR positivity in OSA patients. However, up to date, there are two studies [2, 15] published focused only in the association between nonacid reflux and sleep disordered breathing who concluded that there is no association between non-acid reflux and OSA. Both studies were prospective case control studies, and they presented a statistical significant difference between BMI, in the OSA and non OSA group. According to Halum et al., in obese patients, the correlation with GERD is also quite clear, but the association with LPR alone is not [16]. We believe BMI can act as a confounding variable that could affect the results of association and prediction of LPR in OSA. Our patient had a normal BMI.

Multichannel impedance pH monitoring enables the quantification of acid and non-acid GERD episodes and contact time and also allows the investigators to distinguish liquid-, gaseous-, and mixed-type GERD [17, 18]. It should be done when reflux symptoms remain after prolonged PPI treatment [2, 11]. It may help understanding persistent cough in patients having OSA and GERD [2, 19]

There is no established formal therapy for patients with non-acid GERD. Treatment is usually based on weight loss, lifestyle changes, having small meals, tobacco and alcohol withdrawal, avoiding late dinners, and elevation of the head during bed rest. Prokinetics such as metoclopramide and domperidone, or mucoprotective agents have been shown to be helpful [17, 18]. Therapy with baclofen has also been suggested to improve the tone of the lower esophageal sphincter [18]. Our patient is now stable after taking sodium alginate 500 mg twice a day, and he is currently considering a laparoscopic Nissen fundoplication procedure to be done.

Alginate–antacid formulations may protect from both acid and non-acid gastro-oesophageal reflux [19]. They have proven efficacy for reducing symptoms in GERD, both as and as add-on therapy for patients experiencing breakthrough symptoms with PPIs [20]. Alginate–antacids have a unique mode of action, creating a viscous antacid gel matrix that can form a physical barrier in the proximal stomach that suppresses reflux events. Alginate–antacids also bind pepsin and bile, potentially removing them from the refluxate. This may contribute to the mucosal protection provided by alginates because pepsin is known to damage oesophageal and laryngopharyngeal mucosa even in weakly acidic reflux (pH 5–6) [21].

This is the first reported case documented with DISE where non-acid-type GERD was demonstrated as the primary cause of airway obstruction. We have shown that the epiglottis has reduced its swelling and the AHI has improved significantly after 6 months of medical therapy with alginate. There are countless other potential explanations for the differences between the two sleep studies and the two DISE studies [22, 23]. However, all of them were exhaustively excluded although retrospectively these factors could be confounding ones: there were no concurrent URTI, the patient does not smoke nor is vaping nor in alcohol use. Environmental exposures, allergies, non-allergic rhinitis, fatigue status, hydration level, and other possible confounding factors were carefully excluded [24]. Finally, positional OSA was also excluded, and tongue weakness or glosoptopsis were also excluded.

This novel case presents an interesting theory that deserves attention and may warrant the design of future studies on the association of OSA and GERD, especially when PPI are not effective and non-acid reflux is suspected.

We understand the limited level of evidence of a case report, but this case can orientate the diagnosis in those patients where, despite proper use of PPIs, symptoms remained.

## Conclusions

Thus, non-acid GERD can be a potential cause of OSA in some patients. Medical treatment should be considered before surgery and DISE should be performed in cases where CPAP adaption has failed.

## Data Availability

Not applicable.

## Abbreviations

AHI: Apnea-hypopnea index
BMI: Body mass index
CPAP: Continuous positive airway pressure
DISE: Drug-induced sleep endoscopy
ENT: Ear, nose, and throat
GERD: Gastroesophageal reflux
LPR: Laryngopharyngeal reflux
ODI: Oxygen desaturation index
OSA: Obstructive sleep apnea syndrome
PPI: Proton-pump inhibitor
